# Comparative Analysis of Fecal Bacterial Microbiota of Six Bird Species

**DOI:** 10.3389/fvets.2021.791287

**Published:** 2021-12-08

**Authors:** Li Gao, Li Liu, Chao Du, Qiangchuan Hou

**Affiliations:** ^1^Faculty of Biological Science and Technology, Baotou Teacher's College, Baotou, China; ^2^Hubei Provincial Engineering and Technology Research Center for Food Ingredients, Hubei University of Arts and Science, Xiangyang, China

**Keywords:** gut microbiota, six species, bird, high-throughput sequencing, 16S rRNA gene

## Abstract

The gut microbiota contributes to host health by maintaining homeostasis and improving digestive efficiency. Therefore, identifying gut microbes will shed light on the annual life cycle of animals and in particular those that are threatened or endangered. Nonetheless, the gut microbial composition of the majority of bird species is still unknown. Here, for the first time, 16S rRNA gene sequencing was used to characterize and compare the community composition and diversity of gut microbiotas from six species of birds raised at the Wildlife Conservation Center in Baotou, China: relict gull (*Larus relictus*; *n* = 3), muscovy duck (*Cairina moschata*; *n* = 3), ruddy shelduck (*Tadorna ferruginea*; *n* = 3), demoiselle crane (*Anthropoides virgo*; *n* = 4), whooper swan (*Cygnus cygnus*; *n* = 3), and black swan (*Cygnus atratus*; *n* = 5). A total of 26,616 operational taxonomic units from 21 samples were classified into 32 phyla and 507 genera. Chao1, Shannon diversity, observed species, and Simpson index analysis revealed differences in the community richness and diversity between the different species. Proteobacteria was the dominant bacterial phylum in whooper swan and relict gull, whereas Firmicutes was the dominant bacterial phylum in the other species. At the genus level, 11 dominant genera were detected (*Lactobacillus, Psychrobacter, Enterococcus, Carnobacterium, Weissella, Burkholderia, Escherichia/Shigella, Leuconostoc, Buttiauxella, Desemzia*, and *Staphylococcus*). Principal component and cluster analyses revealed that, while the microbial community composition of different individuals of the same species clustered together, the gut microbial composition varied between the bird species. Furthermore, the most abundant bacterial species differed between bird species. Because many avian gut microbes are derived from the diet, the eating habits and natural living environment of birds may be important contributing factors to the observed differences. Short-term changes to the diet and living environment have little effect on the composition of the avian gut microbiota. This study provides a theoretical basis for bird protection, including disease prevention and control.

## Introduction

The gut microbiome is a collection of all microbial cells and associated genetic material present in the digestive tract of a host. The vital roles of gut microbiota in host metabolism, nutrition, physiology, immune function, and disease resistance are increasingly recognized (1, 2). To date, studies on the gut microbiotas of avian species have focused on the effects of specific bacteria or bacterial pathogens on the host (3, 4). High-throughput sequencing is widely used for quick and efficient characterization of the gut microbial communities of many organisms, including humans (5), cows (6), pigs (7), chickens (8), and a select number of bird species (9–12).

Birds are indicators of the environment and play important ecological roles. Wild birds have naturally adapted to harboring complex gastrointestinal microbial communities comprising hundreds of species at densities as high as 10^11^ colony-forming units per gram (13). The avian gut microbial composition, including the influencing factors, has been analyzed by high-throughput sequencing. For instance, Wu et al. analyzed the gut microbial communities of long-distance migratory swan geese (*Anser cygnoides*) migrating from their breeding area (Khukh Lake in Mongolia) to their wintering area (Poyang Lake in China) and found that the environmental habitat alters the gut microbial community of these birds (14). Zhao et al. analyzed the gut microbial community of hooded cranes (*Grus monacha*) wintering at Shengjin Lake, China (10). Further, Wang et al. analyzed the gut microbial community of black-necked cranes (*Grus nigricollis*) in six wintering areas in China (11), and Wang et al. performed a detailed comparison of gut metagenomes from greylag goose and ruddy shelduck (9).

Characterization of the avian gut microbial community contributes to the knowledge of wild bird microbiology and will likely advance efforts to protect the living environment of these wild birds. Moreover, identification of pathogenic bacteria harbored by wild birds helps prevent the spread of related diseases. However, the gut microbial communities of many species of wild birds have not yet been characterized. Furthermore, eating habits affect the composition of gut microbes (15), but the differences in the gut microbiotas of different species of birds living in the same environment have not yet been studied.

In the current study, the feces of relict gulls (*Larus relictus*; LR), muscovy ducks (*Cairina moschata*; CM), ruddy shelducks (*Tadorna ferruginea*; TF), demoiselle cranes (*Anthropoides virgo*; AV), whooper swans (*Cygnus cygnus*; CC), and black swans (*Cygnus atratus*; CA) raised at the Wildlife Conservation Center of Baotou (Inner Mongolia, China) were sampled. High-throughput sequencing was used to compare the gut microbiotas of these six bird species. The findings of the study contribute to the understanding of gut microbial composition of different species of birds living in the same environment and with access to similar food sources and provide a theoretical basis for bird protection, including disease prevention and control.

## Materials and Methods

### Ethics Statement

The current study was performed in accordance with the recommendations on animal care and ethics in China. Non-invasive techniques were used to collect fecal samples (16). The Animal Ethics and Welfare committee of Baotou Wildlife Conservation Center and Baotou Teachers College approved the implementation of the project.

### Study Objects and Area

The study was designed to compare the gut microbiotas of different species of birds (LR, CM, TF, AV, CC, and CA) from the same living environment and with access to the same food sources. The birds selected for study were raised in the Wildlife Conservation Center of Baotou (Inner Mongolia, China) from July of 2020 to May of 2021. Baotou (109°14′ E to 110°52′ E; 40°23′ N to 41°07′ N) has a semi-arid, mid-temperate continental monsoon climate and is an important stopover site for many migratory birds. The annual average temperature is 7.2 °C, the annual average wind speed is 1.2 m/s, the annual precipitation is 421.8 mm, and the annual sunshine time is 2882.2 h (17).

### Sample Collection

Twenty-one fecal samples from six species of birds were collected at the Wildlife Conservation Center of Baotou: three from relict gulls (*L. relictus*), three from muscovy ducks (*C. moschata*), three from ruddy shelducks (*T. ferruginea*), four from demoiselle cranes (*A. virgo*), three from whooper swans (*C. cygnus*), and five from black swans (*C. atratus*). The birds were housed in the same area and separated in different spaces according to species. The birds had free access to water and food (corn, bran, soybean meal, and alfalfa) and had sufficient space to move around.

To ensure sampling uniformity, all samples were collected in May. Generally, these birds leave feces at sleeping sites. One fecal ball was assumed to come from one bird. Fresh fecal samples were collected in the morning from the feeding space of the different bird species and rapidly transferred to sterile 5-mL centrifuge tubes. To minimize possible contamination from the ground, only the upper layer of a fecal ball was collected. The samples were placed on dry ice, transported to the laboratory, and stored at −80°C until further analysis.

### DNA Extraction

DNA was extracted using the E.Z.N.A. Stool DNA Kit (D4015; Omega Bio-tek, Inc., Norcross, GA, USA) according to the manufacturer's instructions. Total DNA was eluted in 50 μL of elution buffer and stored at −80°C until polymerase chain reaction (PCR) analysis, conducted by LC-Bio Technology Co., Ltd (Hangzhou, China).

### PCR Amplification and 16S rRNA Gene Sequencing

The V3-V4 hypervariable region of the bacterial 16S rRNA gene was amplified using primers 341F (5′-CCTACGGGNGGCWGCAG-3′) and 805R (5′-GACTACHVGGGTATCTAATCC-3′). The reaction mixture contained 25 ng of template DNA, 12.5 μL of PCR Premix, 2.5 μL of each primer, and PCR-grade water to adjust the reaction volume to 25 μL. The PCR amplification conditions were as follows: an initial denaturation step at 98°C for 30 s, followed by 32 cycles of denaturation at 98°C for 10 s, annealing at 54°C for 30 s, extension at 72°C for 45 s, and a final extension step at 72°C for 10 min. The PCR products was confirmed by 2% (w/v) agarose gel electrophoresis. Ultrapure water, instead of the template, was used as a negative control to exclude the possibility of false-positive PCR results. The PCR products were purified using AMPure XT beads (Beckman Coulter Genomics, Danvers, MA, USA) and quantified using a Qubit fluorometer (Invitrogen, Waltham, MA, USA). Amplicon pools were prepared for sequencing, and the size and quantity of the amplicon library were assessed using a 2100 Bioanalyzer system (Agilent Technologies, Santa Clara, CA, USA) and the Library Quantification Kit for Illumina (Kapa Biosciences, Woburn, MA, USA), respectively. The libraries were sequenced using the NovaSeq PE250 platform (Illumina) provided by LC-Bio Technology Co., Ltd., according to the manufacturer's recommendations.

### Data Analysis

Paired-end reads were assigned to samples based on the unique barcodes and truncated by cutting off the barcode and primer sequence. Paired-end reads were merged using FLASH (https://ccb.jhu.edu/software/FLASH/). The raw reads were quality-filtered using specific filtering conditions to obtain high-quality clean reads with fqtrim software (v0.94; https://ccb.jhu.edu/software/fqtrim/). UCLUST (http://drive5.com/usearch/manual/uclust_algo.html) was employed to classify high-quality sequences into operational taxonomic units (OTUs) at an identity threshold of 97% similarity. Chimeric sequences were filtered using Vsearch software (v2.3.4; https://zenodo.org/record/200330). Singleton OTUs (OTUs with only one sequence) were removed from all datasets. Based on the information extracted from the SILVA database (Version 138; https://www.arb-silva.de/documentation/release-138/), each OTU was assigned to the lowest possible taxonomic level on the basis of a minimum bootstrap threshold of 80%. The OTU table was subsampled correspondingly to adjust the sampling depth using the “multiple_rarefactions.py” program from the QIIME pipeline (http://qiime.org/). Alpha- and beta-diversity were calculated based on the *de novo* taxonomic tree constructed using the representative chimera-checked OTU set via FastTree (http://www.microbesonline.org/fasttree/). OTU level α-diversity indices, such as Chao1, Observed species, Shannon index, and Simpson index, were calculated using the OTU table in QIIME to evaluate richness and diversity of bacterial species. Principal component and cluster analyses based on the weighted and unweighted UniFrac distance matrices were performed to determine beta diversity. The linear discriminant analysis (LDA) effect size was evaluated to reveal the significant ranking of abundant modules in six species of samples (18). A size-effect threshold of 2.0 on the logarithmic LDA score was used for discriminative functional biomarkers. Figures were plotted using the R software (19) with “ggplot2” package (https://cloud.r-project.org/web/packages/ggplot2/index.html).

## Results

### Sequencing Statistics

**Figure 1:**
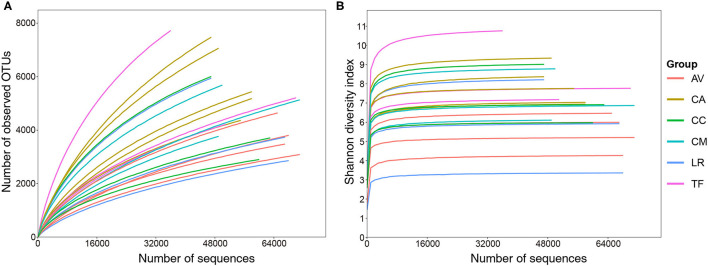
Rarefaction **(A)** and Shannon–Wiener diversity **(B)** curves of bacterial populations in the analyzed samples. AV, demoiselle crane (*Anthropoides virgo*); CA, black swan (*Cygnus atratus*); CC, whooper swan (*Cygnus cygnus*); CM, Muscovy duck (*Cairna moschata*); LR, relict gull (*Larus relictus*); TF, ruddy shelduck (*Tadorna ferruginea*).

Overall, 10,19,740 raw sequence reads of full-length bacterial 16S rRNA were obtained from 21 fecal samples of six species of birds. Between 23,369 and 68,007 (mean: 48,559 ± 12,607) effective sequences (length 400–500 bp) were obtained per sample. A total of 26,616 OTUs at a sequence-similarity level of 97% were identified. These OTUs were classified into 32 phyla, 86 classes, 129 orders, 211 families, and 507 genera. Rarefaction curve analysis revealed that increasing the sequencing depth would allow identification of more OTUs (Figure 1A). However, the value of Shannon-Wiener diversity curves reached saturation (Figure 1B), which indicated that the sequencing depth was sufficient, although other new phylotypes may be identified by further sequencing.

### Alpha Diversity Analysis

**Figure 2:**
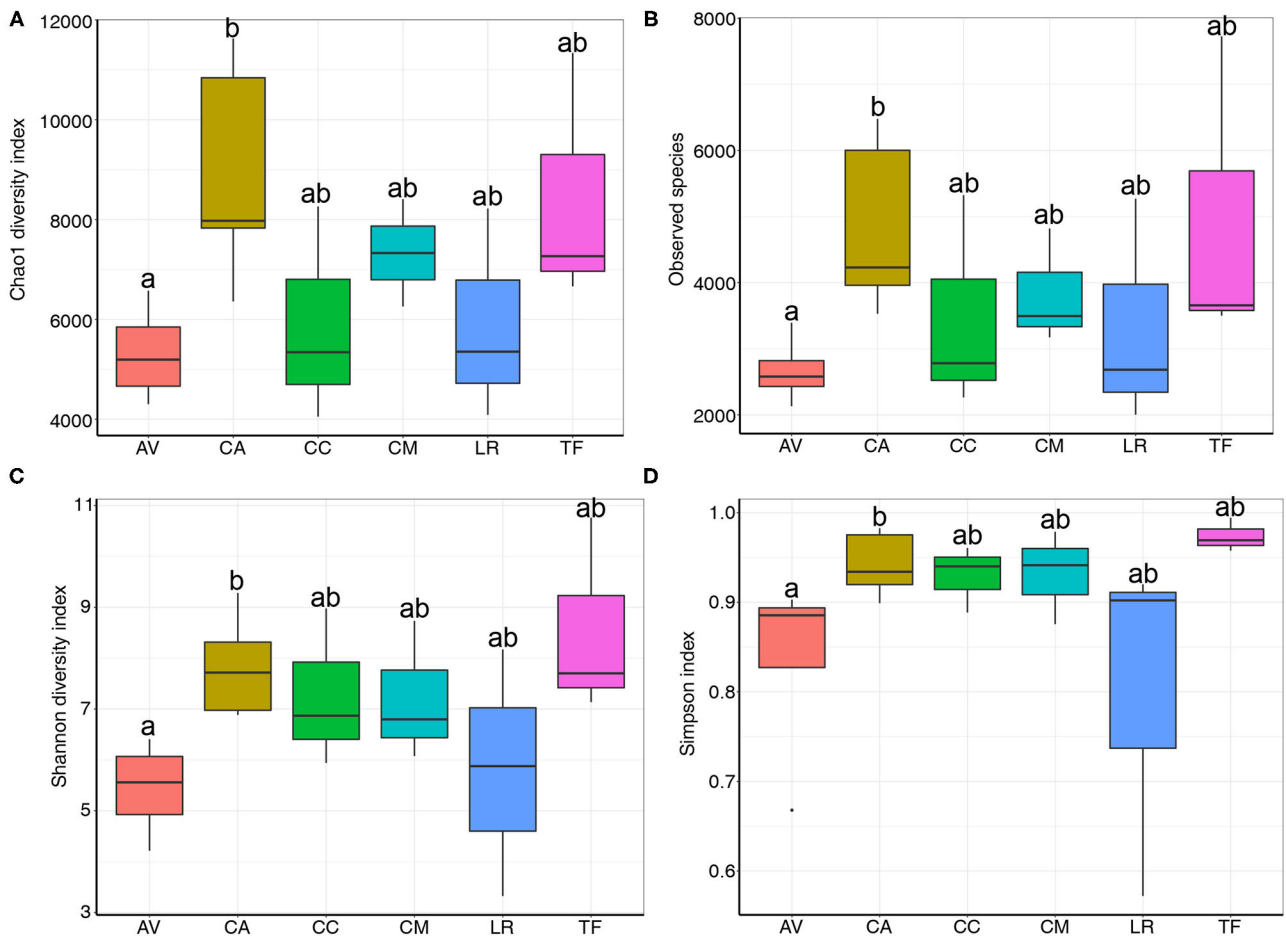
Chao1 diversity **(A)**, observed species **(B)**, Shannon diversity **(C)** and Simpson indices **(D)** of bacterial populations in each sample. AV, demoiselle crane (*Anthropoides virgo*); CA, black swan (*Cygnus atratus*); CC, whooper swan (*Cygnus cygnus*); CM, muscovy duck (*Cairina moschata*); LR, relict gull (*Larus relictus*); TF, ruddy shelduck (*Tadorna ferruginea*).

Rank abundance curve analysis is used to determine the uniformity and richness of species distribution in a sample. Herein, the analysis revealed that the gut microbial composition differed between samples (Supplementary Figure S1). The rank abundance curve of AV was shorter than that of other samples, indicating low bacterial diversity in the AV samples. In contrast, the rank abundance curves of CA and TF were longer than those of the other samples, indicating a relatively high bacterial diversity in these samples. Moreover, Chao1 diversity (Figure 2A), observed species (Figure 2B), Shannon diversity (Figure 2C), and Simpson indices (Figure 2D) all indicated differences in species richness and diversity between bird species. Notably, community diversity differed significantly between the AV and CA samples (Chao1: *p* = 0.037; observed species: *p* = 0.02; Shannon diversity: *p* = 0.02; Simpson index: *p* = 0.037). The differences in community diversity between other species were not significant.

### Global Composition of Gut Bacterial Communities of Six Bird Species

**Figure 3:**
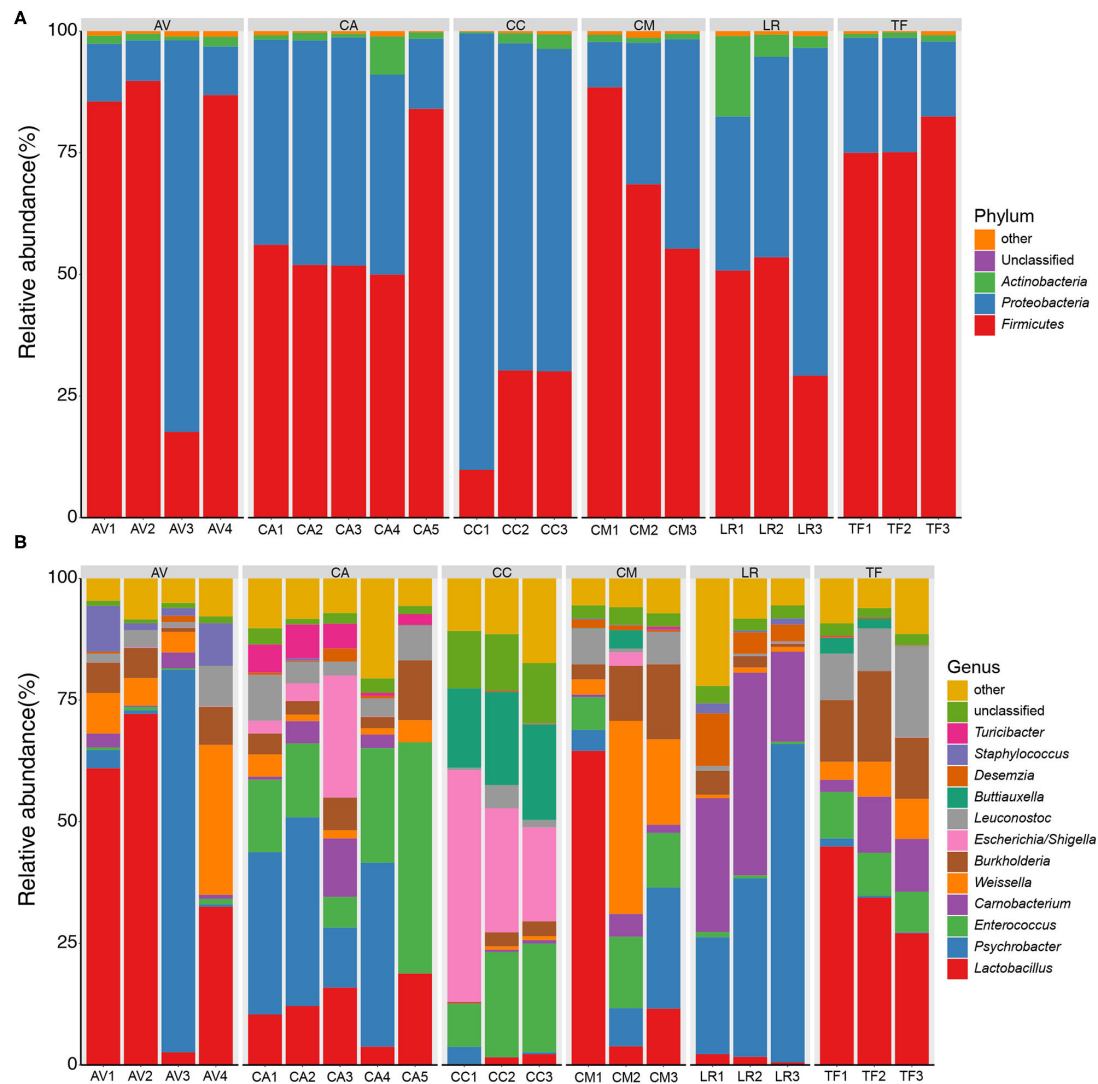
Bar chart of relative bacterial abundance at phylum **(A)** and genus **(B)** level. Bacterial phyla and genus with relative abundance (%) over 1% are shown. Others, bacterial taxa with a relative abundance of ≤1%. AV, demoiselle crane (*Anthropoides virgo*); CA, black swan (*Cygnus atratus*); CC, whooper swan (*Cygnus cygnus*); CM, muscovy duck (*Cairina moschata*); LR, relict gull (*Larus relictus*); TF, ruddy shelduck (*Tadorna ferruginea*).

The bacterial compositions of the fecal samples at the phylum level were analyzed next. A total of 32 bacterial phyla were identified in the 21 fecal samples; three phyla (Firmicutes, Proteobacteria, and Actinobacteria) showed an average relative abundance above 1% (Figure 3A). The cumulative proportion of these three phyla amounted to 98.56% in each sample. The CC and LR gut microbiotas were dominated by Proteobacteria, whereas the dominant phylum in the other bird species was Firmicutes. The bacterial composition of fecal samples at the genus level was also analyzed. A total of 507 bacterial genera were identified in the 21 fecal samples; 11 genera showed an average relative abundance above 1%, namely *Lactobacillus, Psychrobacter, Enterococcus, Carnobacterium, Weissella, Burkholderia, Escherichia/Shigella, Leuconostoc, Buttiauxella, Desemzia*, and *Staphylococcus* (Figure 3B). Further, different genera were dominant in different bird species.

### Shared OTUs in the Gut Microbiotas of Six Bird Species

**Figure 4:**
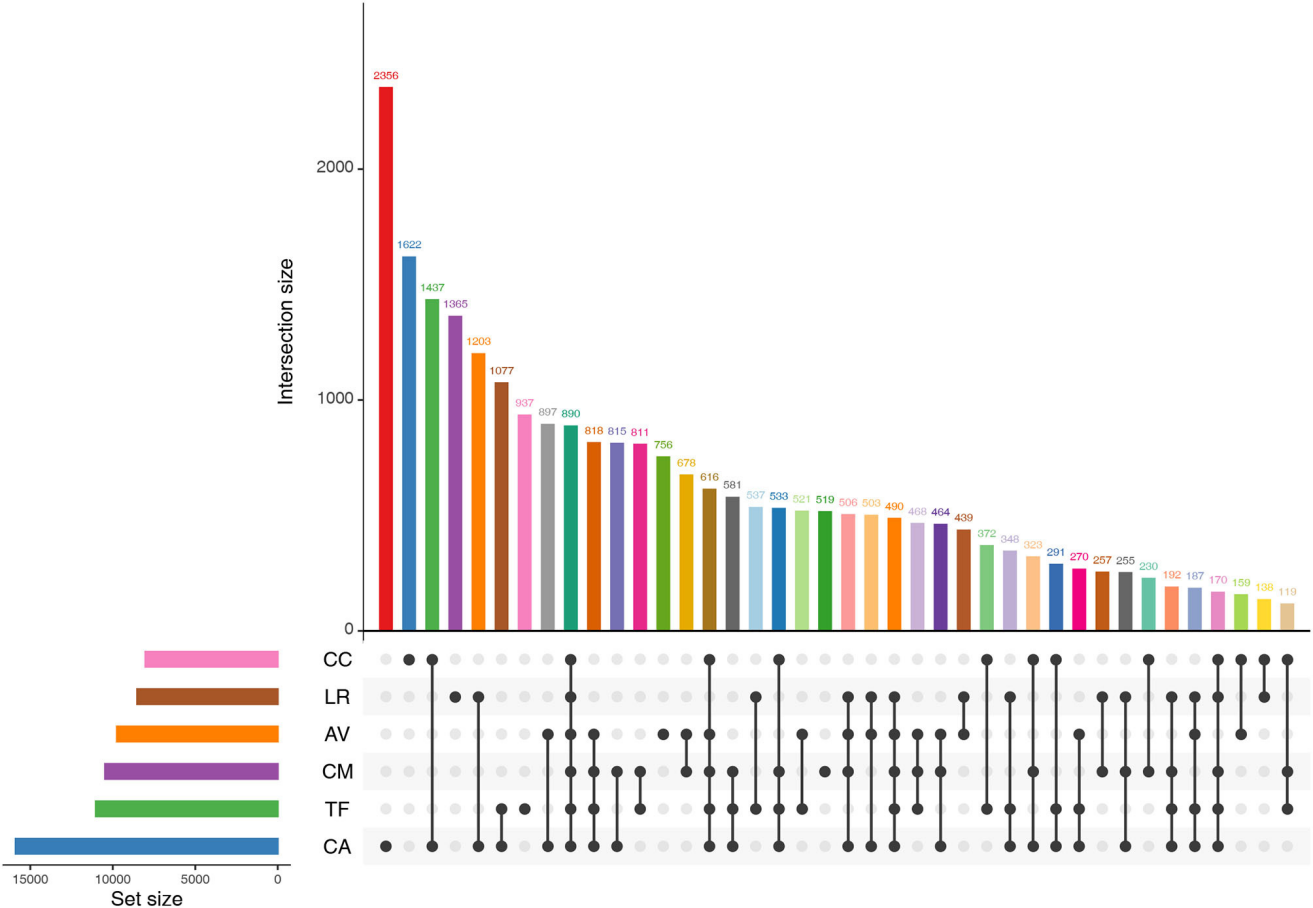
Shared OTUs by the gut microbiota of different bird species. AV, demoiselle crane (*Anthropoides virgo*); CA, black swan (*Cygnus atratus*); CC, whooper Swan (*Cygnus cygnus*); CM, muscovy duck (*Cairina moschata*); LR, relict gull (*Larus relictus*); TF, ruddy shelduck (*Tadorna ferruginea*).

We found that 890 OTUs were shared among the six bird species (Figure 4).The number of sequences contained in these OTUs accounted for 69.96% of the number of sequences sequenced in each sample (range: 45.33–88.30%). The shared OTUs were affiliated mainly with the genera, *Psychrobacter, Lactobacillus, Carnobacterium, Burkholderia*, and *Buttiauxella*.

### Beta Diversity Analysis

**Figure 5:**
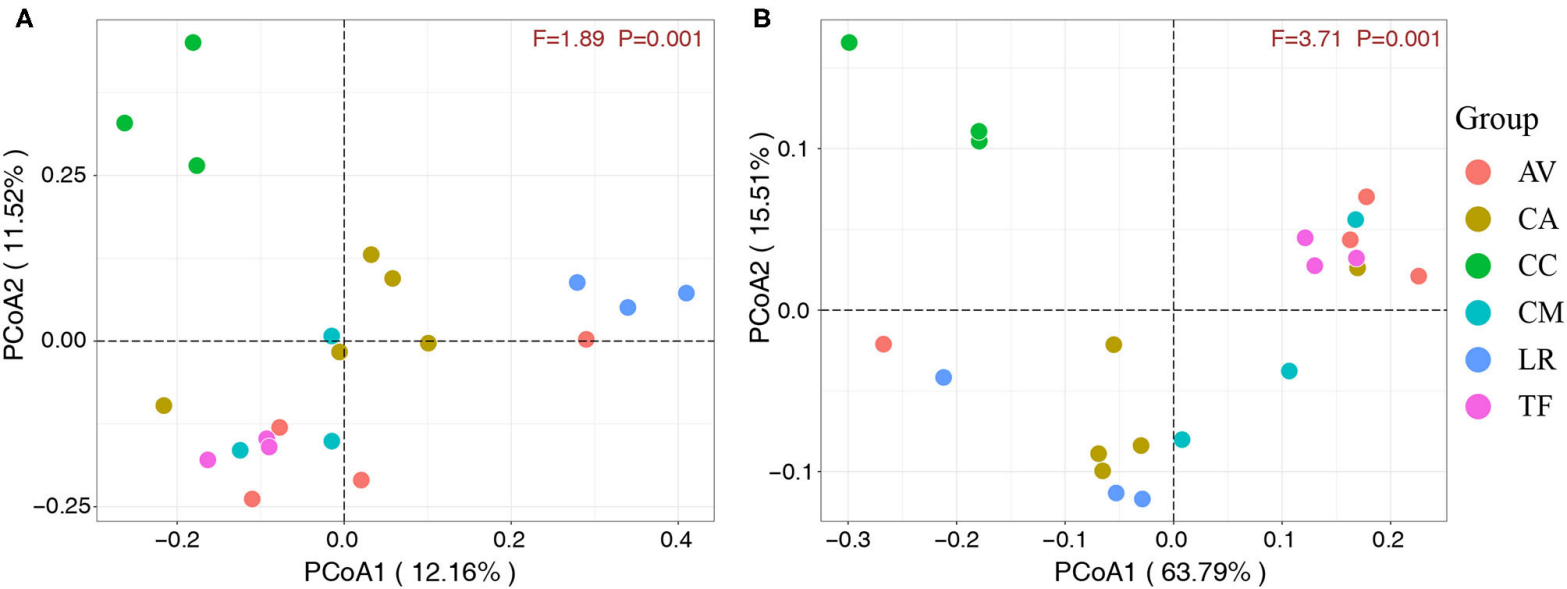
Principal component analysis score plots based on unweighted **(A)** and weighted **(B)** UniFrac distance. Each point represents the mean principal component scores of all samples from one species at one time point. AV, demoiselle crane (*Anthropoides virgo*); CA, black swan (*Cygnus atratus*); CC, whooper Swan (*Cygnus cygnus*); CM, muscovy duck (*Cairina moschata*); LR, relict gull (*Larus relictus*); TF, ruddy shelduck (*Tadorna ferruginea*).

The 21 fecal samples were divided into six groups according to the bird species under investigation. Scatter plots (Figures 5A,B) were generated using the values of the first and second principal components based on unweighted UniFrac distance and weighted UniFrac distance, respectively. The analysis revealed significant clustering of fecal samples from the same bird species, which indicated that the gut microbial compositions among different individuals of the same species were highly similar. Furthermore, the gut microbial composition was similar in the LR and CA groups, while the TF, CM, and AV groups were clustered together and also similar in terms of gut microbial composition. However, the CC group samples clustered separately. These observations indicate that the gut microbial composition of wild birds varies between different species.

### Differences Between Gut Microbial Communities of the Six Bird Species

**Figure 6:**
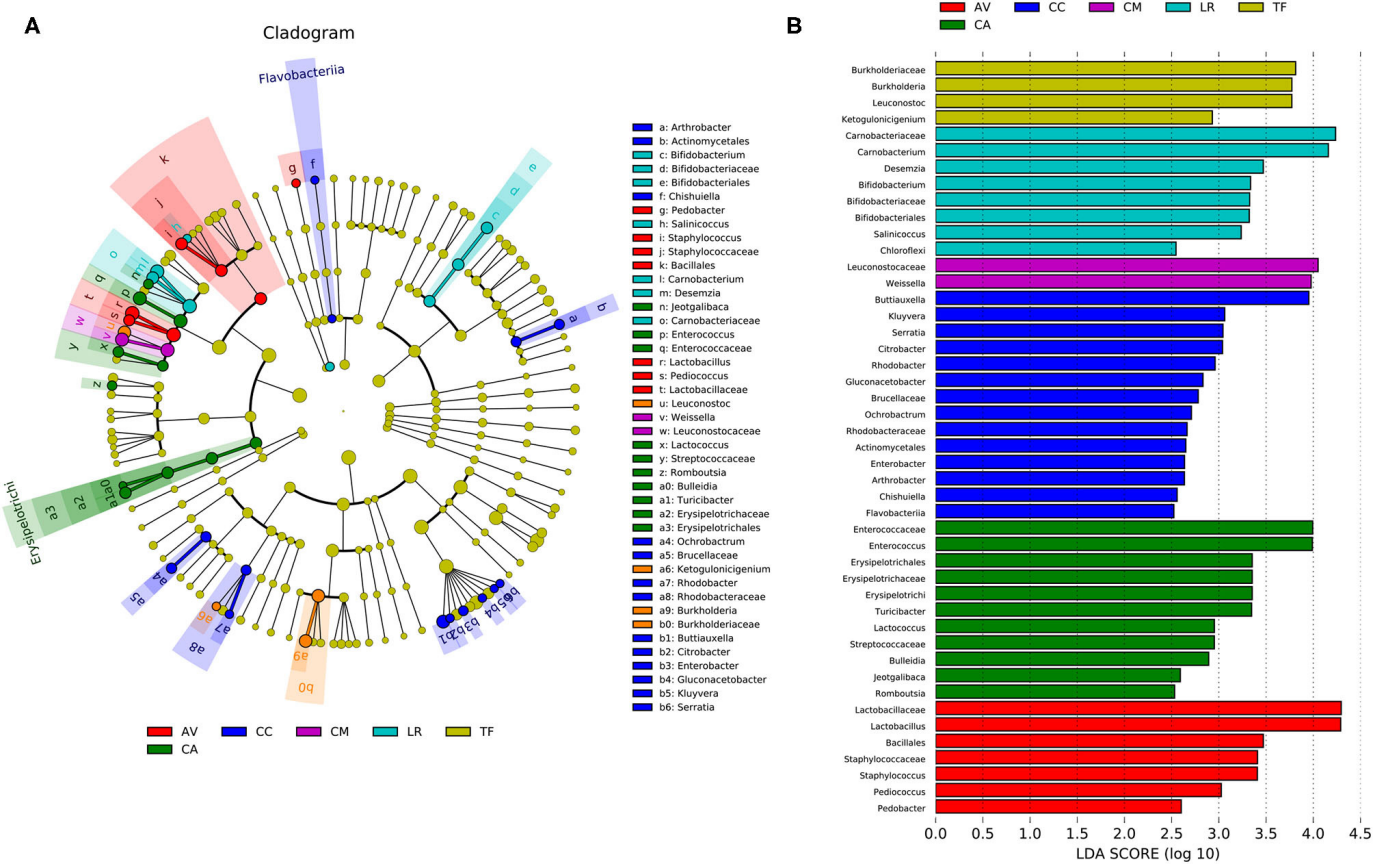
Linear discriminant analysis effect size (LEfSe) analysis. **(A)** The cladogram diagram shows the microbial species with significant differences in the six species of birds. Different colors indicate different groups, with the species classification at the level of phylum, class, order, family, and genus shown from the inside to the outside. **(B)** Plot from LEfSe analysis. The plot was generated using the online LEfSe project. The length of the bar column represents the linear discriminant analysis (LDA) score. The figure shows the microbial taxa with significant differences between the six species of birds (LDA score > 2.0). AV, demoiselle crane (*Anthropoides virgo*); CA, black swan (*Cygnus atratus*); CC, whooper Swan (*Cygnus cygnus*); CM, muscovy duck (*Cairina moschata*); LR, relict gull (*Larus relictus*); TF, ruddy shelduck (*Tadorna ferruginea*).

Analysis of the gut microbial composition of the different bird species revealed substantial differences (Figure 6A). The plot obtained from LDA effect size analysis (Figure 6B) displays the LDA scores of microbial taxa with significant differences among six bird species. In the TF group, the bacteria with significantly higher abundance values than in the other groups were *Burkholderia, Leuconostoc*, and *Ketogulonicigenium*; the abundance of *Carnobacterium, Desemzia, Bifidobacterium, Salinicoccus*, and *Chloroflexi* was significantly higher in LR than in the other groups. *Leuconostocaceae* and *Weissella* were significantly more abundant in the CM group than in other groups. The CC group had more unique flora compared with other groups, including *Buttiauxella, Kluyvera, Serratia, Citrobacter, Rhodobacter, Gluconacetobacter, Arthrobacter, Chishuiella, Flavobacterium*, and *Ochrobactrum*. In the CA group, *Enterococcus, Erysipelotrichi, Turicibacter, Lactococcus, Bulleidia, Jeotgalibaca, Romboutsia*, and some bacteria belonging to *Streptococcaceae*, were significantly more abundant than in the other groups. In the AV group, *Lactobacillus, Staphylococcus*, and *Pedobacter* were significantly more abundant than in the other groups (LDA > 2.0; *p* < 0.05).

## Discussion

The gut microbiota is important for host metabolism, immune function, and disease resistance (1, 2). Imbalances in the gut microbial composition have been associated with various diseases (20). Herein, the gut microbiota of six bird species (LR, CM, TF, AV, CC, and CA) raised at the Wildlife Conservation Center of Baotou (Inner Mongolia, China) were compared, using high-throughput sequencing. Our analyses revealed that the gut microbial composition of the birds, which were fed the same diet and inhabited the same environment, was largely species-dependent. These findings will add to our understanding of the avian gut microbiota, including inter-species differences, as well as provide a theoretical basis for bird protection, including disease prevention and control.

A total of 32 bacterial phyla were identified in 21 fecal samples. Three of these phyla (Firmicutes, Proteobacteria, and Actinobacteria) were present at an average relative content of more than 1%. The average proportion of bacteria from these three phyla in the sequenced data reached 98.56%. The dominant bacterial phyla identified in the current study were consistent with those identified by Wang et al. (21) in wintering whooper swans of Sanmenxia in Henan province and Rongcheng in Shandong province and those identified by Wang et al. (11) in wintering black-necked cranes (11). Therefore, bacteria from these three phyla are dominant in most wild birds analyzed to date. The fourth most abundant phylum in the current study was Bacteroidetes, although its average abundance was 0.34%. In humans, the presence of Firmicutes and Bacteroidetes is an indicator of gut health (22). Hence, our present data may be useful as indicators of intestinal health in the bird species under investigation. However, owing to the differences in the digestive tract of human and birds, whether the birds were healthy or not requires further investigation.

In the present study, 11 genera were identified as dominant in fecal samples from six bird species. Some of these genera (*Lactobacillus, Psychrobacter, Enterococcus, Carnobacterium*, and *Staphylococcus*) have also been identified in other bird species. Lactic acid bacteria identified in birds in this and other studies, including *Lactobacillus, Weissella* (23), and *Leuconostoc* (24), play an important role in regulating intestinal health and may reflect the intestinal health of the birds. However, we also detected some pathogenic bacteria, such as *Burkholderia, Escherichia, Shigella*, and *Ochrobactrum*. Some species of *Burkholderia* cause lung infection (25), while *Escherichia* and *Shigella* are common and potentially fatal intestinal pathogens that cause bacterial food poisoning, typhoid fever, and uremia but are also present in low numbers in the feces of healthy carriers (26). *Ochrobactrum* is a genus in the family of *Brucellaceae* (27) that, despite its reportedly low virulence, is increasingly being associated with infections in immunocompetent hosts, including serious conditions such as endocarditis and septicemia (28, 29). Overall, the present data suggest that relevant measures should be put in place to avoid the spread of diseases via birds.

According to previous studies, dietary composition and living environment affect the gut microbial composition in birds (20, 30). In this study, we observed differences in the gut microbial composition of different bird species. Considering that the diet and living environment of the different bird species in the conservation center were the same, we propose that the observed differences were jointly determined by the host species, different eating habits, and living environment in the wild. The influence of eating habits and living environment in the wild on the gut microbial composition would not completely disappear as a result of short-term changes. It was noted that in particular, *Carnobacterium* was significantly higher in LR compared with the other groups. Previous studies indicated that *Carnobacterium* is widely distributed in the intestines of various fish (31), while fish was an important part of the diet of LR. The relative abundances of *Buttiauxella, Kluyvera, Serratia, Citrobacter, Rhodobacter*, and *Arthrobacter* were significantly higher in CC than in other groups. *Buttiauxella* and *Kluyvera* are frequently isolated from slugs and snails (32). *Serratia* species inhabit the gut of various insect orders, including Hymenoptera, Lepidoptera, Neuroptera, and Hemiptera (33). *Citrobacter* is a common symbiotic taxon among insects and fruit flies specifically (34) and belongs to Gammaproteobacteria, a class that includes dominant symbiotic bacteria in many insect lineages (35, 36). Snails and insects were important parts of the swans' diet. Therefore, the significantly higher relative abundances of *Buttiauxella, Kluyvera, Serratia*, and *Citrobacter* in CC vs. other species may be closely related to CC eating habits. *Rhodobacter* and *Arthrobacter* are common microorganisms in water (37) and soil (38), respectively. Therefore, these results indicate that, although the diet and living environment of birds have a degree of influence on their gut microbial communities, short-term changes to the diet and living environment had little effect on the avian gut microbial composition.

We also observed that the gut microbial composition differed according to species, but this was not the absolute determining factor. For example, whooper swans and black swans belong to the same genus, *Cygnus*, but we observed substantial differences in the gut microbial communities of these two different species, indicating that a close evolutionary relationship is not always associated with a similar gut microbial composition. This may be explained by the observations of Song et al. who reported that, while in non-flying mammals, diet and short-term evolutionary relatedness drive the microbiome composition, and many microbial species are specific to a particular kind of mammal, this pattern is broken in flying mammals and birds, with many microbes shared across different species (39). Therefore, the adaptation to flight may have severed the long-maintained relationship between the host and the microbes.

In summary, we used high-throughput sequencing to analyze fecal samples from six bird species and found that the gut microbiota differed between host species. This difference may be closely related to the dietary habits and living environment of different birds in the wild. Short-term changes in the diet and living environment had little effect on the composition of the gut microbial community. Moreover, the gut microbial composition differed between closely related species. Comparison of gut microbial communities of different bird species will elucidate factors that shape the composition of the gut microbiota in different bird species. The study provides a theoretical basis for bird protection, including disease prevention and control.

## Data Availability Statement

The datasets presented in this study can be found in online repositories. The names of the repository/repositories and accession number(s) can be found below: https://www.mg-rast.org/linkin.cgi?project=mgp99824.

## Ethics Statement

The animal study was reviewed and approved by the Animal Ethics and Welfare Committee (AEWC) of Baotou Wildlife Conservation Center and Baotou Teachers College.

## Author Contributions

LG, LL, and CD designed the study and performed the experiments. LG collected the fecal samples. LG and QH analyzed the data and wrote the manuscript. All authors contributed to the article and approved the submitted version.

## Funding

This work was funded by the High-level Talents Introduced Scientific Research Startup Fund Project of Baotou Teacher's College (No. BTTCRCQD2020-003) and the Inner Mongolia Natural Science Foundation (No. 2021BS08013).

## Conflict of Interest

The authors declare that the research was conducted in the absence of any commercial or financial relationships that could be construed as a potential conflict of interest.

## Publisher's Note

All claims expressed in this article are solely those of the authors and do not necessarily represent those of their affiliated organizations, or those of the publisher, the editors and the reviewers. Any product that may be evaluated in this article, or claim that may be made by its manufacturer, is not guaranteed or endorsed by the publisher.

## Abbreviations

OTU: operational taxonomic unit
PCR: polymerase chain reaction
LR: *Larus relictus*
CM: *Cairna moschata*
TF: *Tadorna ferruginea*
AV: *Anthropoides virgo*
CC: *Cygnus cygnus*
CA: *Cygnus atratus*.
