# Supplementary figures and images for: Comparative Analysis of Fecal Bacterial Microbiota of Six Bird Species
Source: Front Vet Sci. 2021 Dec 8;8:791287. doi: 10.3389/fvets.2021.791287 (PMC8692710; doi:10.3389/fvets.2021.791287)

# Supplementary Material

## Supplementary Figures

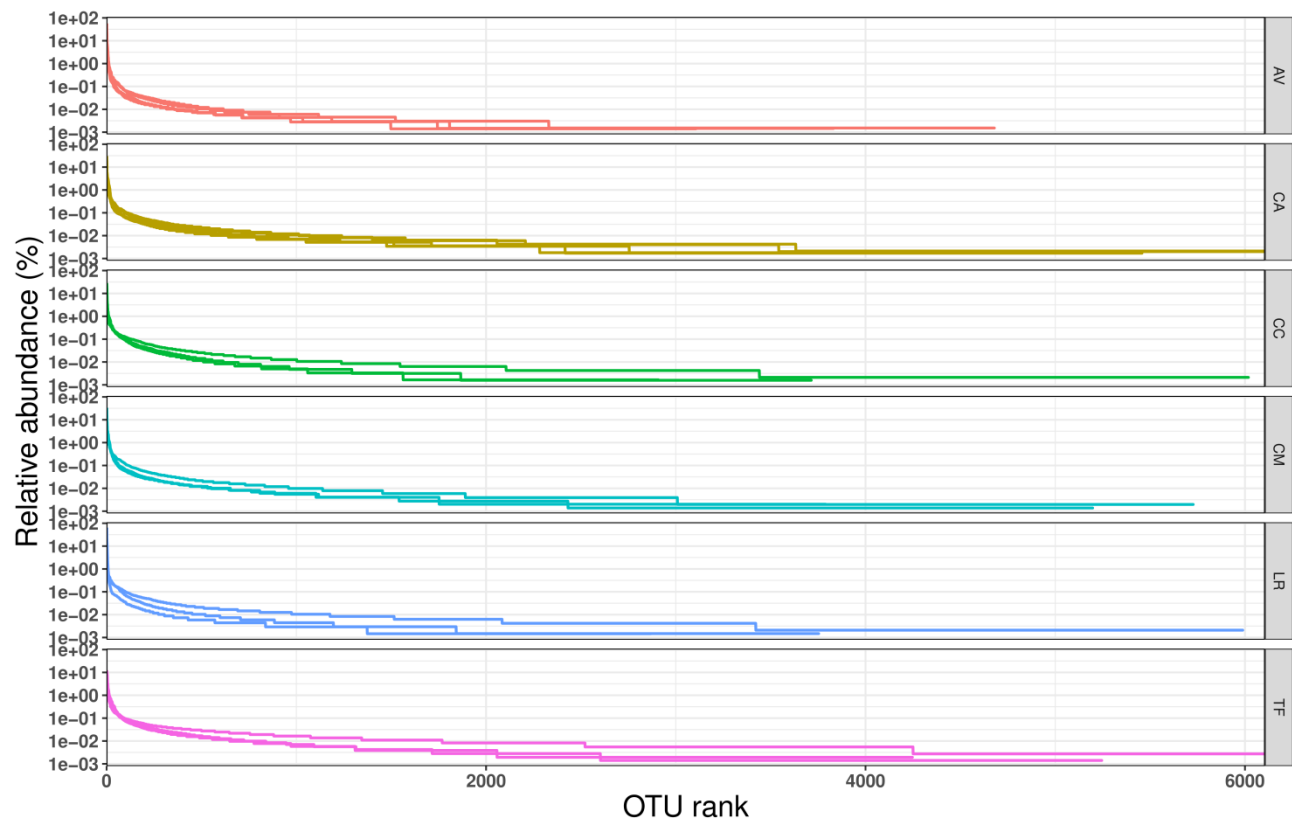

**Supplementary Figure 1.** Rank abundance curve.

Supplement: Supplementary file 1 [file Image_1.pdf]
